# The complete chloroplast genome sequence of *Hippeastrum rutilum* (*Amaryllidoideae*)

**DOI:** 10.1080/23802359.2020.1820388

**Published:** 2020-09-23

**Authors:** Baohua Huang

**Affiliations:** Department of Food and Horticulture, Zhangzhou Institute of Technology, Zhangzhou, PR China

**Keywords:** *Hippeastrum rutilum*, ornamental plant, complete chloroplast genome

## Abstract

*Hippeastrum rutilum* is one of the world’s important ornamental plants. Here, we report the complete chloroplast genome of *H. rutilum* by whole-genome data. The whole cp genome is 158,357 bp in length, consisting of two inverted repeat regions (IR, 26,817 bp), one large single-copy region (LSC, 86,451 bp), and one small single-copy region (SSC, 18,272 bp). A total of 133 genes annotated for the chloroplast genome, including 87 protein-coding genes, 38 tRNAs, and eight rRNAs. Phylogenetic analysis pointed to that *H. rutilum* was closely related to *Narcissus poeticus* and *Lycoris radiata.*

*Hippeastrum rutilum* is a perennial herb in the *Amaryllidoideae* family, which is native to Brazil. *H. rutilum* is also an important ornamental plant all over the world. The flowers of *H. rutilum* are beautifully shaped and brightly colored. Now it is widely cultivated in tropical, subtropical areas. However, its phylogenetic relationships are rather limited. In this study, we reported the complete chloroplast genome sequence of *H. rutilum*, which would be helpful for its genetic and evolutionary research.

The sample of *H. rutilum* was collected from the Longhai County, Zhangzhou City, China, located at 24°11′∼24°36′N and 117°29′∼118°14′E. The specimen was stored at specimen room of Zhangzhou Institute of Technology (specimen NO. ZITP2019830). The genomic DNA of *H. rutilum* was extracted from leaves by plant genomic DNA kit (Tiangen Biotech, Beijing, China) and sequenced using the Illumina Novaseq platform (Illumina, San Diego, CA). The cp genome was assembled by the GetOrganelle (Jin et al. 2018) and then annotated with the Geseq (Tillich et al. 2017) online software with Manual correction. Finally, a complete chloroplast genome of *H. rutilum* was obtained and submitted to GenBank with the accession number MT133568 and the link is https://www.ncbi.nlm.nih.gov/nuccore/MT133568.

The complete cp genome of *H. rutilum* is 1,58,357 bp in length, containing a large single-copy (LSC) region of 86,451 bp, a small single-copy (SSC) region of 18,272 bp, and two inverted repeat (IR) regions of 26,817 bp. The new sequence has 133 genes in total, including 87 protein-coding genes, 38 *tRNA* genes, and eight *rRNA* genes. In addition, the overall GC content of the genome is 37.93%, whereas the corresponding ratios of the LSC, SSC, and IR regions are 54.59, 11.54, and 33.87%, respectively.

In order to investigate its phylogenetic position, other 10 complete cp genomes of *Amaryllidaceae* species (KT630835, MN200196, NC_045534, NC_035971, NC_044709, NC_039825, NC_045077, NC_030708, NC_030712, and NC_025593) were aligned with *H. rutilum* using MAFFT (Katoh and Standley 2013). A maximum likelihood analysis was performed by RAxML (Stamatakis 2014) with 1000 bootstrap replicates. The results showed that *H. rutilum* was closely related to *Narcissus poeticus* and *Lycoris radiata* (Figure 1).

**Figure 1.**
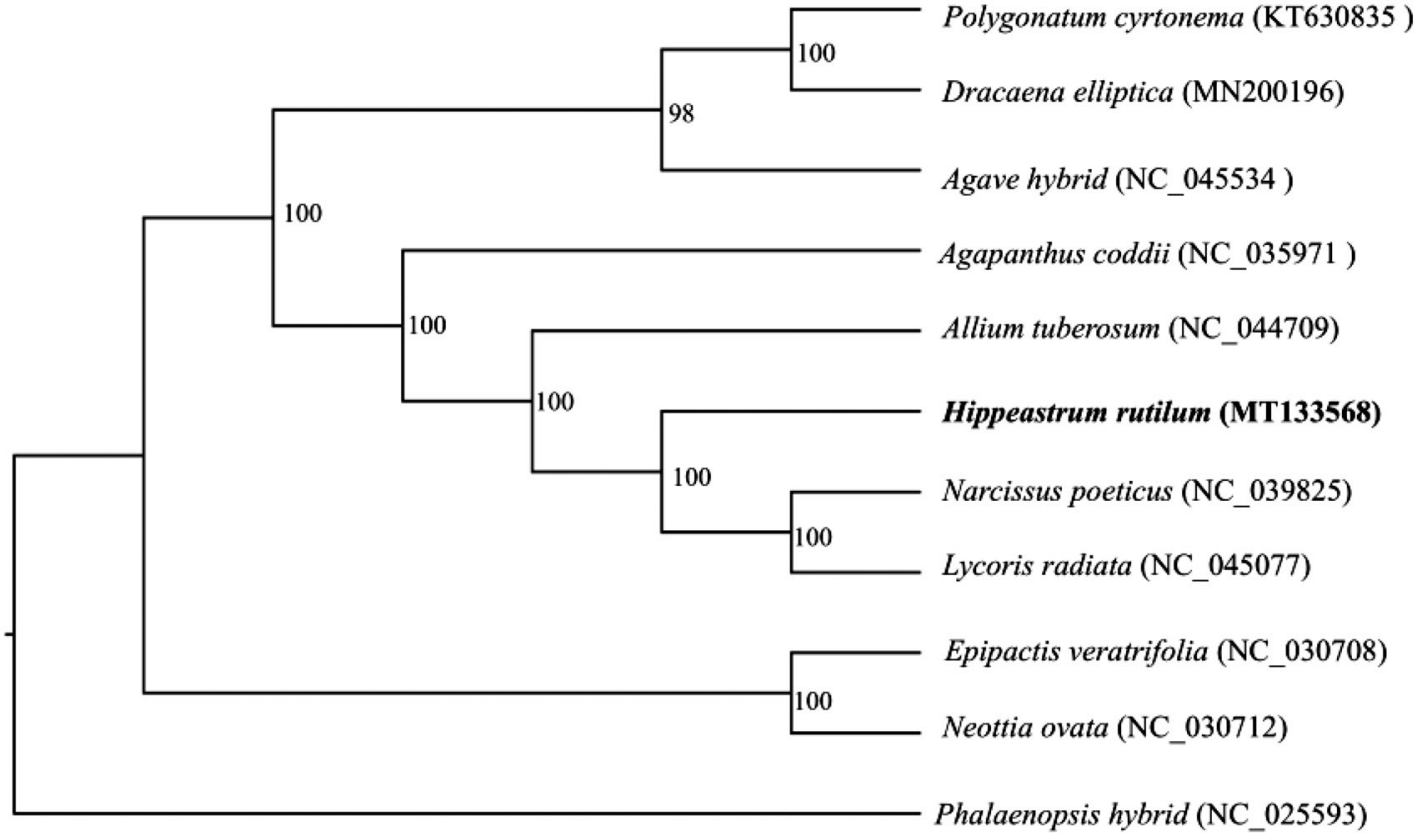
Maximum likelihood tree of *H. rutilum* and other Amaryllidoideae species based on whole chloroplast genome sequences, with *Phalaenopsis hybrid* as the outgroup. Bootstrap support values (based on 1000 replicates) are shown next to the nodes.

## Data Availability

The data that support the findings of this study are openly available in GenBank with the Accession number MT133568, the link is https://www.ncbi.nlm.nih.gov/nuccore/MT133568. Raw data were generated at the Geseq online software with Manual correction. The data and materials supporting the results or analyses presented in their article freely available. The data should only be shared if it is ethically correct to do so, where this does not violate the protection of human subjects, or other valid ethical, privacy, or security concerns. The data that support the findings will be available in GenBank from the date of publication to allow for commercialization of research findings. The data supporting the results of this study and the derived data can be obtained from the corresponding author H B H on reasonable request. The sample of *H. rutilum* was collected from the Longhai County, Zhangzhou City, China. It is located at 24°11′∼24°36′N and 117°29′'∼118°14′E.

## References

[CIT0001] Jin JJ, Yu Wen-Bin Y, Jun-Bo Y, Yu S, Ting-Shuang Y, De-Zhu L. 2018. GetOrganelle: a fast and versatile toolkit for accurate de novo assembly of organelle genomes. bioRxiv. 256479. 10.1186/s13059-020-02154-5 PMC7488116 32912315

[CIT0002] Katoh K, Standley DM. 2013. MAFFT multiple sequence alignment software version 7: improvements in performance and usability. Mol Biol Evol. 30(4):772–780. 23329690 10.1093/molbev/mst010 PMC3603318

[CIT0003] Stamatakis A. 2014. RAxML version 8: a tool for phylogenetic analysis and post-analysis of large phylogenies. Bioinformatics. 30(9):1312–1313. 24451623 10.1093/bioinformatics/btu033 PMC3998144

[CIT0004] Tillich M, Lehwark P, Pellizzer T, Ulbricht-Jones ES, Fischer A, Bock R, Greiner S. 2017. GeSeq - versatile and accurate annotation of organelle genomes. Nucleic Acids Res. 45(W1):W6–W11. 28486635 10.1093/nar/gkx391 PMC5570176

